# The Clinical Frailty Scale can be used retrospectively to assess the frailty of patients with hip fracture: a validation study

**DOI:** 10.1007/s41999-022-00686-6

**Published:** 2022-08-20

**Authors:** Robert S. Kay, Martin Hughes, Thomas R. Williamson, Andrew J. Hall, Andrew D. Duckworth, Nick D. Clement

**Affiliations:** 1 Scotland Foundation School, Royal Infirmary of Edinburgh, Edinburgh, UK; 2 Edinburgh Medical School, University of Edinburgh, Edinburgh, UK; 3 Department of Orthopaedics, Golden Jubilee National Hospital, Clydebank, UK; 4 Department of Orthopaedics & Trauma, University of Edinburgh, Edinburgh, UK; 5 Edinburgh Orthopaedics, Royal Infirmary of Edinburgh, Edinburgh, UK

**Keywords:** Frailty, Validation, Hip fracture, Rockwood, Retrospective

## Abstract

**Purpose:**

Frailty is a common clinical syndrome affecting hip fracture patients. Recognising and accurately assessing frailty status is important in clinical and research settings. The Rockwood Clinical Frailty Scale (CFS) is a commonly used instrument and demonstrates a strong correlation with mortality and length of hospital admission following hip fracture. What is not understood, however, is the validity of retrospectively assigned CFS scores in hip fracture patients. The aim of this study was to assess the validity of retrospective non-orthogeriatrician assigned CFS scores in hip fracture patients.

**Methods:**

Hip fracture patients from a single major trauma centre were assessed and CFS scores were assigned prospectively by non-orthogeriatric clinicians (*n* = 57). A subset of these patients were also assigned a prospective CFS score by a specialist orthogeriatrician (*n* = 27). Two separate blinded observers (non-orthogeriatric clinicians) assigned CFS scores retrospectively using electronic patient records alone. Agreement and precision was examined using the Bland–Altman plot, accuracy was assessed using *R*^2^ statistic and inter-rater reliability was assessed using quadratic weighted Cohen’s kappa.

**Results:**

Seventy percent of the cohort were female with an average age of 83. Agreement was high between prospective non-orthogeriatrician assigned CFS scores and retrospective non-orthogeriatrician assigned CFS scores, with a low bias (0.046) and good accuracy (*R*^2^ = 73%). Good agreement was also seen in comparisons between prospective orthogeriatrician assigned CFS scores versus retrospective non-orthogeriatrician assigned scores, with a low bias (0.23) and good accuracy (*R*^2^ = 78%). Good inter-rater reliability was seen between blinded observers with a quadratic weighted Cohen’s kappa of 0.76.

**Conclusions:**

Retrospective CFS scores assigned by non-orthogeriatricians are a valid means of assessing frailty status in hip fracture patients. However, our results suggest a tendency for non-orthogeriatricians to marginally overestimate frailty status when assigning CFS scores retrospectively.

**Level of evidence:**

3.

## Key summary points

**Aim:**

The aim of this study was to assess the validity of retrospective non-orthogeriatrician assigned CFS scores in hip fracture patients.

**Findings:**

Retrospective CFS scores assigned by non-orthogeriatricians are a valid means of assessing frailty status in hip fracture patients.

**Message:**

Our findings confirm the validity of retrospectively assigned CFS scores in hip fracture patients for use in clinical and research settings.

## Introduction

Frailty is a common clinical syndrome characterised by reduced physiological reserve, impaired cognition and an increased predisposition to adverse healthcare outcomes [1]. The construct of frailty was first introduced 3 decades ago as a means of understanding and defining the complex health status of older adults [2]. While chronological age and comorbidity are inherently related to frailty, these factors are distinct and, therefore, careful consideration must be given when assessing frailty status [3]. Currently there is no universally established definition of frailty [1], although various scoring systems have been designed to capture frailty status in clinical and research settings.

Numerous tools for measuring and reporting frailty exist. These scoring systems can be broadly categorised as multidimensional assessments, physical performance-based instruments or judgement-based scoring systems [4]. The Rockwood Clinical Frailty Scale (CFS) is one of the most commonly used systems in the United Kingdom [2]. It is a judgement-based ordinal scoring system that assigns a score of one (least frail) to nine (most frail) based on the patient’s mobility, independence, cognition and symptom burden. A written criteria for each score and a visual aid is provided to guide the user to generate an accurate score. In the inpatient setting, the CFS is intended to reflect the patients’ general health status one month prior to admission as an indication of pre-morbid health. As many as 40% of hip fracture patients demonstrate some form of cognitive impairment [5], therefore, the CFS offers a pragmatic means of assessing frailty in the hip fracture population.

Hip fracture is the most common acute orthopaedic presentation in the UK with over 70,000 hip fractures recorded annually [6]. In hip fracture patients CFS is a powerful predictor of mortality, length of hospital admission and return to domicile [7, 8]. CFS on admission demonstrates greater discriminative ability in predicting mortality compared to chronological age or Association of Anesthesiologists (ASA) grade [7, 9]. Furthermore, frailty has been demonstrated to increase the risk of sustaining future fractures due to a multitude of factors including increased falls susceptibility [10] and reduced bone mineral density [11]. It is, therefore, pertinent to identify the presence and degree of frailty in hip fracture patients to better inform inpatient management, such as level of geriatric input, prognostication and communication of risk to patients and relatives.

Retrospective assignment of CFS from patient records for use in clinical audit and research has become an increasingly common practice [12–15]. The validity of retrospective CFS assignment has been demonstrated by various authors in acute medical patients [16–18]. However, there is a paucity of data to prove the validity of retrospective non-orthogeriatrician (non-OG) assigned CFS scores in orthopaedics populations.

The primary aim of this study was to assess the agreement, precision, accuracy and reliability of prospective non-OG assigned CFS score versus retrospective non-OG assigned CFS score in hip fracture patients. Secondary aims were to investigate the agreement between prospective orthogeriatrician (OG) assigned CFS score (expert user) versus retrospective non-OG assigned CFS score, as well as to examine retrospective inter-rater agreement, precision, accuracy and reliability.

## Methods

### Patient recruitment

**Fig. 1:**
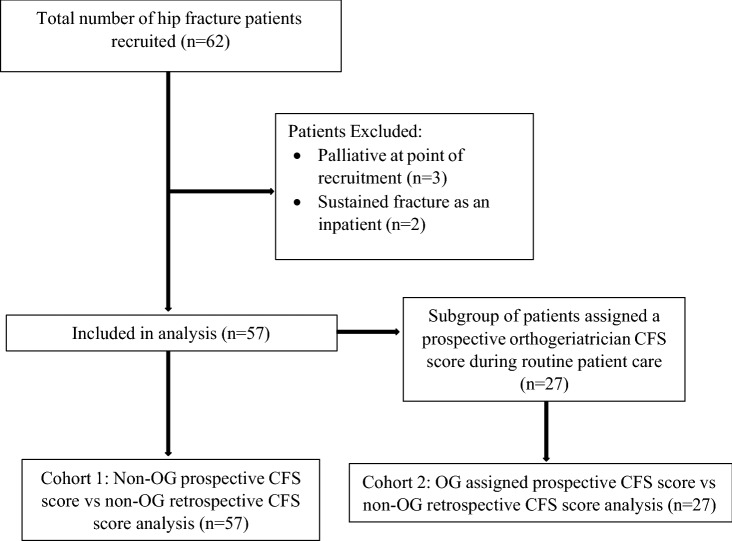
Study population flow diagram

All patients over 50 that were admitted with an acute hip fracture to the Royal Infirmary of Edinburgh during three study periods between November 2021 and January 2022 were included in the study (*n* = 62). The patient recruitment cycles were undertaken approximately 3 weeks apart to capture a consecutive cohort of new patient admissions. Patients who sustained a hip fracture as an inpatient (ambiguity over pre-morbid CFS status in such patients) or were considered end-of-life (large amount of subjectivity over CFS score 9—‘terminally ill’) were excluded. Following exclusions two cohorts were defined prior to analysis. Cohort 1 included all patients (*n* = 57). Cohort 2 was a subgroup derived from Cohort 1, including all patients who had been assigned a CFS score by a specialist orthogeriatrician (OG) during admission (*n* = 27) see Fig. 1.

### Assignment of CFS

At the point of enrolment a prospective CFS score was assigned by a non-OG. A subgroup of patients were assigned a second prospective CFS score by an OG (blinded to the non-OG CFS score) during frailty assessment as part of routine patient care. The selection of patients within this subgroup was determined by which patients were assessed by orthogeriatric services during admission. Two blinded retrospective CFS scores were subsequently assigned remotely using electronic patient records alone by non-OGs.

### Statistical analysis

Continuous variables were expressed as means and compared using a Student’s *t* test, with standard deviation (SD) or 95% confidence intervals (CI) given where appropriate. Data were tested for normality using a Shapiro–Wilk test, and *p* values < 0.05 were considered statistically significant.

Two assessments of the validity of retrospectively assigned CFS score were undertaken: (1) non-OG retrospective CFS score versus prospective non-OG CFS score (*n* = 57), and (2) non-OG retrospective CFS score versus prospective OG CFS score (*n* = 27). The mean of the retrospective CFS scores from the two blinded raters was used for comparisons. Inter-rater reliability was examined through comparison of the two separately attained blinded retrospective CFS scores.

Agreement was examined using the Bland–Altman plot to compare the difference in CFS score assigned. This method defines bias as the average difference between scores and generates 95% limits of agreement (1.96 × the standard deviation of the bias). Good agreement was defined a priori as 95% confidence intervals < 1 CFS point and bias < 0.5. Further assessment of bias was undertaken using linear regression analysis to examine whether bias was constant or proportional to the mean CFS score.

Precision was examined by calculating the 25th and 75th percentiles of the bias, with interquartile range representing the degree of precision. Accuracy between the prospective and retrospective CFS scores was examined using *R*^2^, with values > 50% considered moderate accuracy and values > 70% considered strong accuracy. Lastly, inter-rater reliability was examined using quadratic weighted Cohen’s Kappa, where values greater than 0.80 were considered to demonstrate a high degree of reliability.

All statistical analysis was undertaken using IBM SPSS 25.0.0.2 software package (SPSS Inc., Chicago IL, USA). Study design and reporting followed guidance from the Standards for Reporting Diagnostic accuracy (STARD) 2015 statement [19].

### Ethics

The study was registered and approved under our departmental orthopaedic research database (Scotland B Research Ethics Committee 20/SS/0125) and the study was also prospectively registered with the musculoskeletal quality improvement committee. The data collection was carried out in accordance with the GMC guidelines for good clinical practice and the Declaration of Helsinki.

## Results

### Study cohort characteristics

The study cohort consisted of a total of 57 patients. Forty (70%) were female and 17 (30%) male, with an overall mean age of 83 years old. There were 29 (51%) intracapsular, 21 (37%) intertrochanteric, 4 (7%) periprosthetic and 3 (5%) subtrochanteric hip fractures. Thirty-two (56%) were left sided and 25 (44%) right sided.

### Prospective non-OG assigned CFS score versus retrospective non-OG assigned CFS score

**Fig. 2:**
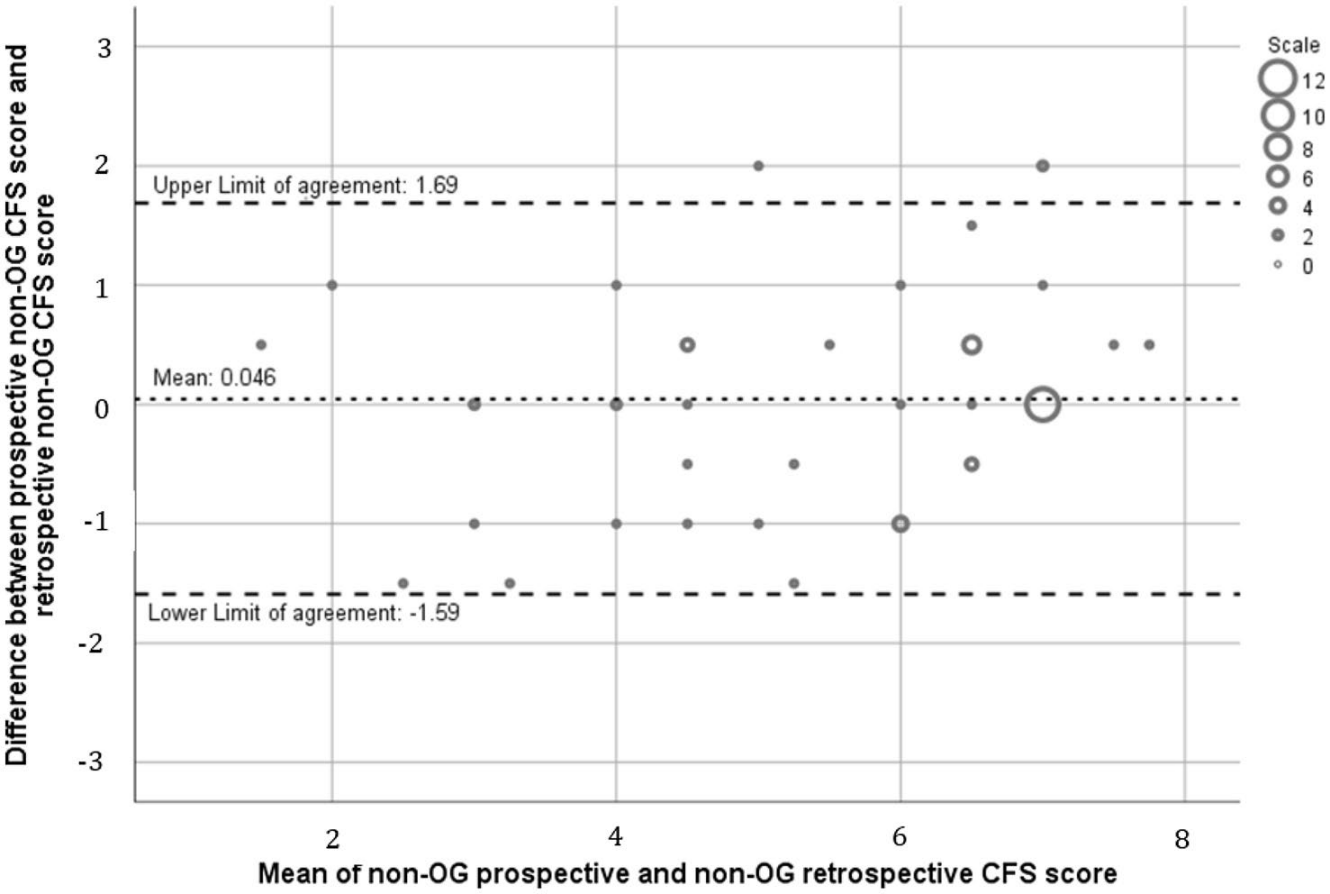
Bland–Altman comparing prospective non-OG versus retrospective non-OG CFS scores (*n* = 57). The dashed lines indicate the upper and lower limits of agreement (1.96 × standard deviations of the bias) and the dotted line indicates mean/bias (0.046)

The mean prospective non-OG CFS score was 5.53 (SD 1.84) while the mean non-OG retrospective CFS score was 5.62 (SD 1.51) with no significant difference detected (*p* = 0.52). Agreement between prospective and retrospective CFS scores was high with a bias of 0.046 (95% confidence intervals of − 0.18 and 0.27). As seen in Fig. 2, the line of equality falls within the 95% limits of agreement suggesting there was no systematic difference. This slight positive bias suggests a marginal overestimation of frailty by retrospective observers. Linear regression identified that there was no proportional bias, with a correlation coefficient of 0.119, (*p* = 0.108). Accuracy as measured by *R*^2^ was high at 73%. The 25th and 75th percentiles of the bias were − 0.5 and 0.5, respectively, suggesting good precision.

### Prospective OG assigned CFS score versus retrospective non-OG assigned CFS score

**Fig. 3:**
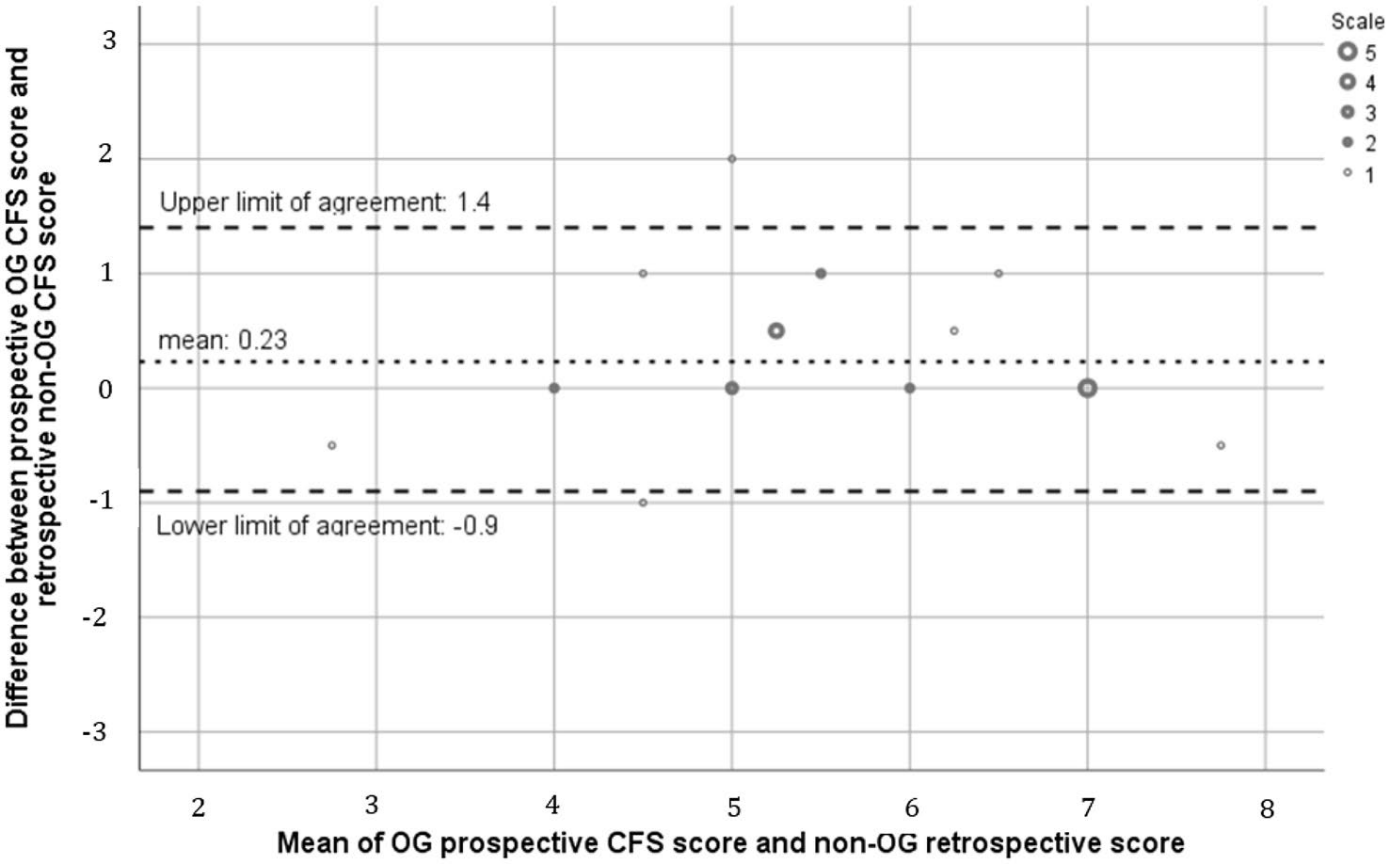
Bland–Altman comparing prospective OG versus retrospective non-OG CFS score (*n* = 27). The dashed lines indicate the upper and lower limits of agreement (1.96 × standard deviations of the bias) and the dotted line indicates the mean/bias (0.23)

The mean prospective geriatrician assigned CFS score was 5.44 (SD 1.54), while retrospective non-OG assigned mean CFS score was 5.62 (SD 1.51) (*p* = 0.08). Agreement between the prospective and retrospective CFS scores was high with a bias of 0.23 and 95% confidence intervals of − 0.03 and 0.49. As seen in Fig. 3, the line of equality falls within the 95% limits of agreement suggesting there is no significant systematic difference. This slight positive bias suggests a marginal overestimation of CFS score by the non-OG retrospective observers. Linear regression identified there was no proportional bias, with a correlation coefficient of 0.284 (*p* = 0.253). Accuracy was considered high with an *R*^2^ statistic of 78%. The 25th and 75th percentiles of the bias were 0 and 0.5, respectively, suggesting good precision.

### Retrospective inter-rater reliability

**Fig. 4:**
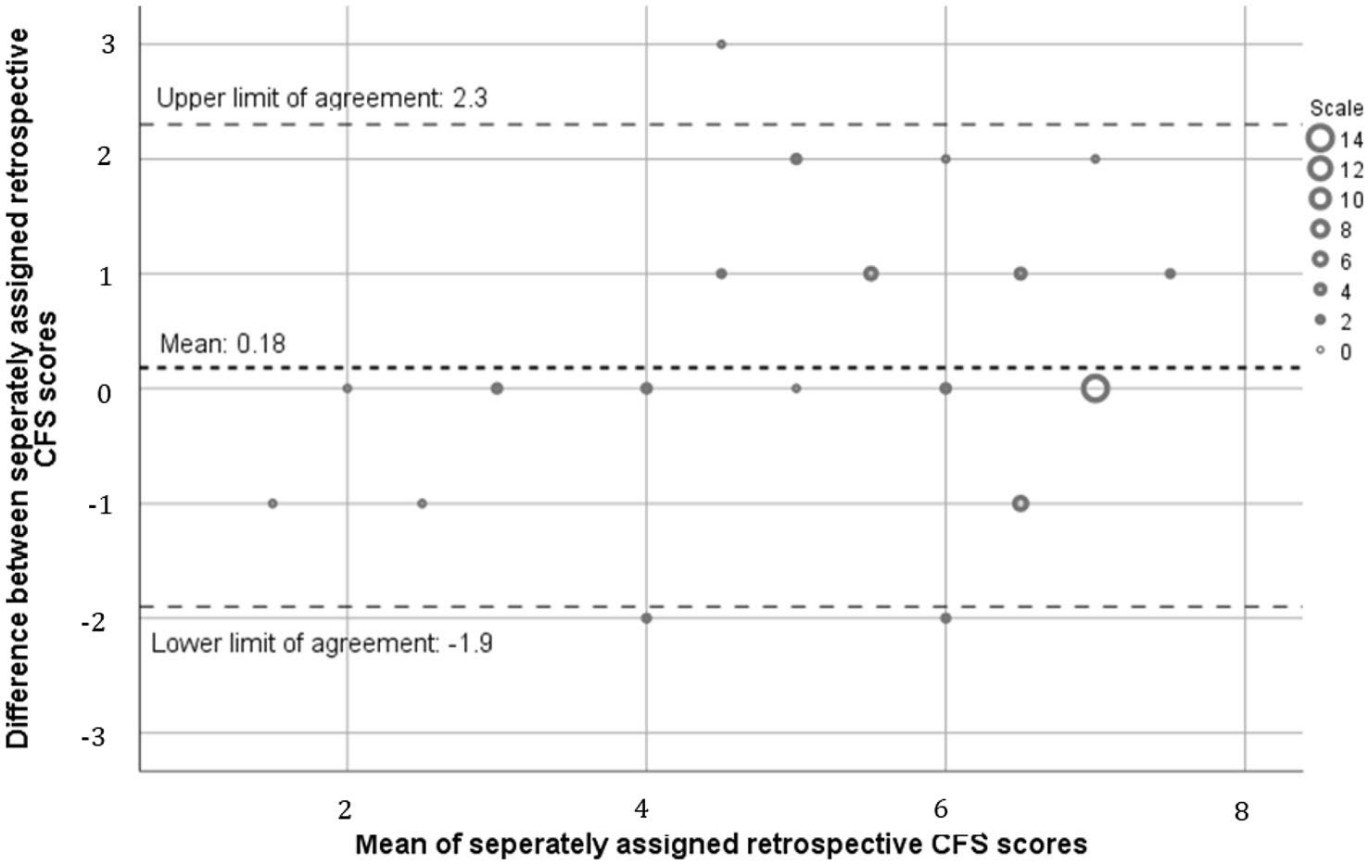
Bland–Altman plot comparing inter-rater reliability between retrospective non-OG raters (*n* = 57). The dashed lines indicate the upper and lower limits of agreement (1.96 × standard deviations of the bias) and the dotted line indicates mean/bias (0.18)

The mean CFS scores assigned by the two blinded observers were 5.75 (SD 1.63) and 5.48 (SD 1.64), with no statistically significance difference (*p* = 0.108). Agreement was high, with a low bias of 0.18 and 95% confidence intervals of − 0.11 and 0.47 (see Fig. 4). Precision was good, with a low interquartile range of 1 (25th quartile 0, 75th quartile 1) and moderate accuracy with an *R*^2^ of 59%. Quadratic weighted Cohen’s Kappa was 0.76 which represents good inter-rater reliability between the two scorers.

## Discussion

This study examined the validity of retrospective use of the Rockwood Clinical Frailty Scale (CFS) by non-specialist clinicians in the assessment of frailty in hip fracture patients. There was good accuracy, precision and agreement between CFS scores assigned prospectively and retrospectively, and good inter-rater reliability between retrospective assessors. This supports the hypothesis that retrospectively assigned CFS scores are a valid means of assessing frailty status in orthopaedic patients admitted with a hip fracture, and these findings are relevant to clinical and research applications of the CFS.

Although there was good agreement between CFS assessments, and bias was well within the pre-specified range of acceptance (95% Cis of < 1 CFS point), there was a tendency for retrospective non-OG observers to marginally overestimate CFS score compared scores assigned prospectively by an expert user. This difference could have been due lack of geriatric experience, lack of understanding of the Rockwood CFS score, or due to differences in assigning CFS score in the ward environment versus using electronic notes alone. This could also be attributed to the fact that the retrospective non-OG raters knew they were being observed and, therefore, the small difference seen may be an artefact of the study design. It is also possible this bias could be accounted for by random variation, since the magnitude of difference was small (0.23). A further direction for this work would be to examine the agreement between blinded specialist geriatrician assigned CFS scores to understand what level of bias one would expect between blinded expert observers.

The present study adopted a novel approach to examine the validity of retrospectively applied CFS assessments by comparing the scores of non-expert users with the scores assigned on prospective clinical examination by expert users (specialist orthogeriatric clinician), which served as a gold standard assessment. Through comparison against a gold standard prospective geriatrician assigned score, we can say with a high degree of certainty that retrospective non-OG CFS score assignment is a valid means of assessing frailty in hip fracture patients. This study design was adopted to reduce observer bias and improve the validity of our findings.

The findings of this study are consistent with other evidence available in the literature [16–18]. While various means of assessing agreement have been used in previous studies, to our knowledge, Stille et al. are the only other authors who have used the Bland–Altman plot to examine agreement between prospective and retrospective CFS scores [17]. The current study findings replicated those of Stille et al, whose analysis of 110 patients in acute care in Germany demonstrated a bias figure of 0.26 and 25th and 75th quartiles of 0 and 1. The current study builds on existing evidence insofar as it assessed the validity of retrospective and non-expert application of the CFS in the context of an orthopaedic trauma population that is older and exhibits a higher prevalence of cognitive impairment.

These findings are relevant to research and clinical practice. There is an established body of clinical research in which frailty-related outcomes have been investigated based upon the assumption that retrospectively assigned CFS scores are a valid means of assessing frailty [12–14]. Retrospective assignment of CFS score has been used in studies investigating outcomes such as mortality and admission duration in patient with hip fracture [7, 15]. The current study indicates that retrospective assignment of a CFS score using patient medical records is a valid technique by which to assess clinical frailty in hip fracture patients. The findings are relevant in clinical practice, where an accurate assessment of frailty is important in guiding the management of hip fracture patients, but where clinical evaluation may not be timely, feasible, or sufficient. Patients identified as being frail may be prioritised for specialist multi-disciplinary team care, such enhanced perioperative support and input from orthogeriatric, physiotherapy and occupational therapy services, all of which have been shown to improve outcomes following hip fracture [20]. Increasing the ability of clinical teams to assess frailty appropriately will enable services to meet the complex care needs of this vulnerable patient group.

Most often patients are initially assessed by a non-OG, it is, therefore, important for non-OGs to make an accurate assessment of frailty at this stage to ensure patients receive the appropriate care based upon their pre-admission frailty.

There are several limitations to the current study. First, the majority (70%) of the study cohort were female, however, this is generally representative of the gender preponderance seen in hip fracture patients. Second, only 27 out of the 57 were assessed by an OG and assigned a prospective OG CFS score. The selection of this subset may introduce bias as OGs are likely to assess patients with certain characteristics including frailty and comorbidity. Lastly, the findings of this study are relevant to inpatients with acute hip fracture and are, therefore, not necessarily generalisable to other patient groups.

Rockwood Clinical Frailty Scale scores assigned retrospectively by non-orthogeriatricians are a valid means of assessing frailty in patients admitted with hip fracture. Agreement, precision, and accuracy were high, and consistent with previous studies examining the validity of retrospectively assigned CFS scores in other clinical contexts.
